# Evaluation of a health promotion program in children: Study protocol and design of the cluster-randomized Baden-Württemberg primary school study [DRKS-ID: DRKS00000494]

**DOI:** 10.1186/1471-2458-12-157

**Published:** 2012-03-06

**Authors:** Jens Dreyhaupt, Benjamin Koch, Tamara Wirt, Anja Schreiber, Susanne Brandstetter, Dorothea Kesztyüs, Olivia Wartha, Susanne Kobel, Sarah Kettner, Dmytro Prokopchuk, Verena Hundsdörfer, Melina Klepsch, Martina Wiedom, Sabrina Sufeida, Nanette Fischbach, Rainer Muche, Tina Seufert, Jürgen Michael Steinacker

**Affiliations:** 1 Institute of Epidemiology and Medical Biometry, Ulm University, Schwabstr. 13, D-89075 Ulm, Germany; 2 Section Sports and Rehabilitation Medicine, University Hospital Ulm, Steinhövelstr. 9, D-89070 Ulm, Germany; 3 Institute for Psychology and Pedagogy, Ulm University, Albert-Einstein-Allee 47, D-89081 Ulm, Germany

## Abstract

**Background:**

Increasing prevalences of overweight and obesity in children are known problems in industrialized countries. Early prevention is important as overweight and obesity persist over time and are related with health problems later in adulthood. "Komm mit in das gesunde Boot - Grundschule" is a school-based program to promote a healthier lifestyle. Main goals of the intervention are to increase physical activity, decrease the consumption of sugar-sweetened beverages, and to decrease time spent sedentary by promoting active choices for healthy lifestyle. The program to date is distributed by 34 project delivery consultants in the state of Baden-Württemberg and is currently implemented in 427 primary schools. The efficacy of this large scale intervention is examined via the Baden-Württemberg Study.

**Methods/Design:**

The Baden-Württemberg Study is a prospective, stratified, cluster-randomized, and longitudinal study with two groups (intervention group and control group). Measurements were taken at the beginning of the academic years 2010/2011 and 2011/2012. Efficacy of the intervention is being assessed using three main outcomes: changes in waist circumference, skinfold thickness and 6 minutes run. Stratified cluster-randomization (according to class grade level) was performed for primary schools; pupils, teachers/principals, and parents were investigated. An approximately balanced number of classes in intervention group and control group could be reached by stratified randomization and was maintained at follow-up.

**Discussion:**

At present, "Komm mit in das Gesunde Boot - Grundschule" is the largest school-based health promotion program in Germany. Comparative objective main outcomes are used for the evaluation of efficacy. Simulations showed sufficient power with the existing sample size. Therefore, the results will show whether the promotion of a healthier lifestyle in primary school children is possible using a relatively low effort within a school-based program involving children, teachers and parents. The research team anticipates that not only efficacy will be proven in this study but also expects many other positive effects of the program.

**Trial registration:**

German Clinical Trials Register (DRKS), DRKS-ID: DRKS00000494

## Background

Overweight and obesity in childhood are common problems in most industrialized countries for a long time [1]. Data from Germany show an overweight prevalence of 15.4% in children aged 7-10 years, and of 18.6% in children aged 11-14 years [2]. At present, this prevalence remains constant in some parts of Germany [3] as well as in other countries (e.g. Greece [4], Sweden [5]). The relatively short observation periods, however, may not allow ensuring consistency of this trend.

There are various reasons for the rising prevalence of overweight and obesity in children. Besides genetic predisposition [6], lifestyle and behavioral factors need to be considered: little physical activity [7,8], unfavorable nutrition (first of all high caloric food [9-11]), sedentary activities in leisure time, e.g. TV/video watching or using PC/play-stations [12]. Furthermore, the cultural and socio-economic background may play a role in the development of overweight and obesity in children as children with migration background and children who grow up in economically or socially disadvantaged families are more often affected by overweight and obesity [13-16].

Childhood overweight and obesity has been shown to affect physical health [17,18] as well as psychosocial aspects [19]. Efforts to treat them are troublesome and in the long run often ineffective [20].

Overweight and obesity persist over time within childhood [21,22] until adulthood [23,24]. Besides a higher risk of cardiovascular, and metabolic diseases, overweight and obesity in children is related with further consequences [22], e.g. reduced quality of life [19], poorer level of academic achievement [25], decreased self esteem [26]. Thus, prevention of overweight and obesity in children are an important public health issue in most industrialized countries.

Thus, randomized-controlled trials of overweight prevention programs with sufficient statistical power and suitable outcome measures are still needed [27,28]. Possible reasons for missing evidence of efficacy may be inappropriate methods, too short intervention periods, methodological problems (e.g. lack of randomized controlled study design), unsuitable outcome variables [29].

The implementation of childhood prevention programs in schools is promising [30-33]. Possible barriers are low, so that all children can be reached easily. Children spend the main part of their day in schools, and teachers are the personnel qualified in dealing with children as well as considering their age-appropriate needs and competencies. Although from a public health perspective the role of schools for health promotion is very important, high demand in time and resources may impede long term implementation of intervention programs. Furthermore, despite the central role of schools, the familial and socio-economic background of children should not be neglected: there are indications that prevention programs which involve parents are more effective than other programs [34].

The program "Komm mit in das gesunde Boot - Grundschule" (Join the Healthy Boat - Primary School) funded by the Baden-Württemberg Stiftung (Baden-Württemberg foundation) is based on promising experiences of the precursor project "URMEL-ICE" ("Ulm Research on Metabolism, Exercise, and Lifestyle Intervention in Children" [15,35]). URMEL-ICE was a randomized controlled study to investigate the efficacy of an overweight prevention program in children. It was a school-based project implemented in 64 classes (2^nd ^grade) in 32 schools in Ulm and adjacent regions in Germany. URMEL-ICE was a one year intervention with focus on health-promoting behavior change towards an increase in physical activity, a decrease in the consumption of sugar-sweetened beverages, and a decrease in time spent with screen media [16]. As main result of URMEL-ICE, positive effects in the intervention group compared to the control group were observed: statistically significant improvements in children's aerobic endurance [Brandstetter S, Koch B, Berg S, Fritz M, Galm C, Klenk J, Peter R, Prokopchuk D, Steiner R, Wartha O, Wabitsch M, Steinacker JM: A randomized controlled trial on school-based overweight prevention - effects on aerobic endurance, but not on motor skill, Submitted] and tendencies for lower increases in measures of body fat (waist circumference and subscapular skinfold thickness) [16].

The program "Komm mit in das gesunde Boot - Grundschule" includes in addition to the project URMEL-ICE all four levels of primary school and is now distributed on large scale to school teachers in the whole state of Baden-Württemberg by special educated project delivery consultants. The main goal of the present study is the evaluation of the program's intervention in a routine application in large scale. In the following, the program and the Baden-Württemberg Study are characterized in more detail.

## Methods/Design

### Program overview

The program "Komm mit in das gesunde Boot - Grundschule" is a health promotion program for primary school children in Baden-Württemberg, South West Germany, which started in 2009. It is developed, implemented and evaluated by a research team at Ulm University together with a team of experienced teachers. The program supplements the curriculum for primary schools focusing on subjects covering "humans", "nature" and "culture". It is a school-based program to promote healthy lifestyle choices in children, to increase physical, mental, and emotional abilities and, consecutively, to attenuate the increase in body fat and thus to prevent overweight and obesity.

The contents of the program are fully integrated in the school environment: the intervention of the program is implemented in school lessons and during recess (For details see section "Intervention"). A system of particular interested teachers (project delivery consultants) was established to spread the program throughout the state of Baden-Württemberg: it is a federal state of Germany which is situated in South West Germany and has an area of about 35.751 km^2 ^with a population of 10.750.000. All project delivery consultants were trained intensively and thoroughly in the program's concept and materials. Based on these central training sessions, these teachers are able to hold local training courses in their region for further primary school teachers. 30 and 34 primary school teachers were trained and certified as project delivery consultants in 2009/2010 and 2010/2011, respectively. These project delivery consultants recruited and trained 453 and 437 primary school teachers in 2009/2010 and 2010/2011 respectively. The project delivery consultants are reimbursed for her/his work; school teachers receive travel subsidies for training lessons and perform the program as part of their routine work.

The advantage of the system is that teachers are trained by a colleague or other teacher and not by an external expert. This enhances acceptance of the program and facilitates translation into the school environment and enables a higher sustainability [36]. Furthermore, such systems provide local training courses which support participation of the primary school teachers because of short distances. Dissemination is facilitated in a whole state approach with limited resources because the scale-cost effects are much lower than in expert intervention programs [37].

The quality of the program is permanently assessed by process evaluation. Additionally, an investigation of effects of the intervention is being performed in the Baden-Württemberg Study. The study protocol was developed in 2009 and approved by the ethics committee of Ulm University in June 2009 (Application No. 126/10). The study started in 2010 with baseline measurements. Follow-up measurements were carried out in 2011. Furthermore, approval of the Baden-Württemberg Study was obtained from the Ministry of Culture and Education of the state of Baden-Württemberg. The teachers and principals of the participating schools as well as the children's parents gave written informed consent to participate. The Baden-Württemberg Study is registered at the German Clinical Trials Register (DRKS), Freiburg University, Germany, under the DRKS-ID: DRKS00000494.

### Aims of the Baden-Württemberg Study

Three parameters are specified as main efficacy outcomes in the study protocol: change in waist circumference, change in subscapular skinfold thickness and change in endurance performance during a 6-min run; changes are measured as difference between follow-up measurement and baseline measurement. These parameters were chosen because of their reasonably objective measurement and the results of the URMEL-ICE study [16]. Changes in a number of parameters concerning the following topics are regarded as further outcomes as well as influencing factors:

• Children's physical, mental, and emotional "fitness"

• Health related quality of life in children

• Behavior and behavior-related cognition in children and parents

• Children's physical activity behavior

• Socio-demographic parameters of children and parents

• Familial and social anamnesis of children and parents

• Education

• School Environment

• Health-economic aspects

The parameters for the further outcomes were chosen because of its importance for the children's health. Details of the parameters are described in the section "Assessments of the Baden-Württemberg Study".

### Study design

**Figure 1:**
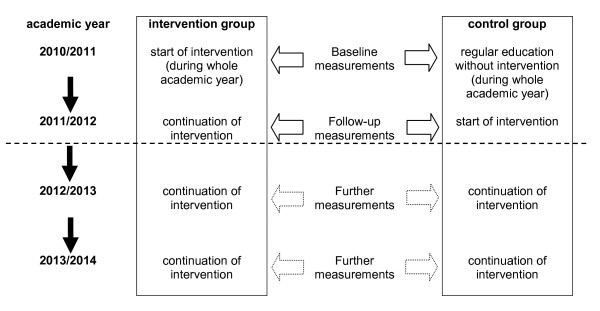
**Overview and time course of the Baden-Württemberg Study**.

Figure 1 shows an overview and the time course of the Baden-Württemberg Study.

The Baden-Württemberg Study is a prospective, stratified, cluster-randomized, and longitudinal study consisting of an intervention group and a control group. The waiting control group design was realized in this study: in the academic year 2010/2011, the program's intervention was carried out in the intervention group whereas the control group followed the regular curriculum (without intervention). In the academic year 2011/2012, the control group started with the intervention, i.e. after one year time of waiting. Cluster-randomization was performed for participating primary schools in Baden-Württemberg. Blinding is not feasible in such a school based study. Measurements were performed at three different levels, namely level 1 "pupil", level 2 "parents", and level 3 "teacher and principal", see section "Data Collection". Participating children are 1^st ^grade and 2^nd ^grade pupils.

Measurements were taken at two points:

• First point: Baseline measurements were taken at the start of the academic year 2010/2011 in the period from mid September 2010 to mid November 2010 (i.e. from the end of summer vacation until the fall vacation in Baden-Württemberg in 2010)

• Second point: Follow-up measurements were taken at the start of the academic year 2011/2012 in the period from mid September 2011 to mid November 2011 (i.e. from the end of summer vacation until the fall vacation in Baden-Württemberg in 2011)

Pupils at randomized primary schools whose parents gave written consent for participation in the study were measured at the two points mentioned above.

Additionally, in November 2010 (baseline measurement) and November 2011 (follow-up measurement) a parental questionnaire was issued and returned within six weeks. Teachers and principals were also asked about characteristics of the school environment during follow-up.

A continuation of the Baden-Württemberg Study is planned as an observational study: pupils taking part in the study will be observed until they finish primary school.

### Actual status of study

At present (winter 2011), all follow-up measurements have been completed. In the next months, data management (scanning of case report form pages and verification of scanned data) and data checks will be performed before starting the statistical analyzes.

### Intervention

The intervention was developed by a team of scientists in collaboration with experienced primary school teachers and is developed for all levels of primary school (i.e. 1^st ^grade until 4^th ^grade). It combines elements from behavioral prevention and situational prevention. The three main goals of the intervention are to increase physical activity, to decrease the consumption of sugar-sweetened beverages, and to decrease time spent with screen media. The intervention follows a saluto-genetic, competence, and action oriented approach and is informed by Social Cognitive Theory [38]. The program's intervention is based on one academic year as part of lesson plans and recess. No additional physical education lessons are necessary to carry out the intervention but physical activity is integrated in the whole school day and in the school environment.

The intervention is implemented by the class teacher. For a progressive consolidation of the intervention's main goals from the 1^st ^grade until 4^th ^grade, a spiral curriculum is promoted: main topics are recessed from grade to grade. The intervention's rationale and contents are included in folders which contain the manual and materials for the intervention (1^st ^and 2^nd ^grade [39], 3^rd ^and 4^th ^grade [40]). Each class teacher belonging to the intervention group has taken part in a three-part local training course. The intervention itself includes 20 units per school year regarding the topics "beverages", "physical activities", and "recreational activities". These units are spread over the whole academic year. Furthermore, the intervention consists of two physical activity exercises which are performed every school day ("active breaks", each exercise takes between 5 and 7 minutes). Additionally, "family homework" exercises are given which are small tasks relating to the lesson's topics to involve the parents. Further, samples for parents' evenings and templates for letters to parents in three languages, i.e. German, Turkish and Russian, are included in the folder.

In the academic year 2010/2011, there was no intervention in the control group: although interested, the class teachers belonging to the control group received no local training and no materials for the intervention: they were registered for the participation in the academic year 2011/2012. In the academic year 2011/2012, these class teachers started with the three-part local training course (waiting control group design, see section "Study Design").

### Quality assurance and process evaluation

Process evaluation is ongoing to assess the quality of central training sessions for project delivery consultants and local training courses for school teachers. To further develop and improve the program, a process evaluation mainly using questionnaires, assessing implementation of and satisfaction with the given materials as well as structure and contents of the training courses is being carried out. Furthermore, information about work load when using the teaching materials and of the basic facilities of participating schools were gathered.

A scientific advisory board was established to support the research team and to help ensure the long-term quality of the program which meets regularly and reviews reports, study plans and results (see appendix).

### Data collection

The collection of data in the Baden-Württemberg Study is realized at three different levels:

• level 1 "pupil": measurement of anthropometric parameters, motor abilities, general well-being and body image was conducted in the total sample; computer-based test for attention and executive function as well as objective measurements of physical activity were performed in two subsamples because of organizational and technical reasons, see section "Study Sample".

• level 2 "parents": a self-constructed questionnaire for parents was used to measure parameters like socio-economic background, health behaviors and other related components.

• level 3 "teacher and principal": information about education and school environment was assessed.

### Assessments of the Baden-Württemberg Study

In the following section, details of all assessments used in the Baden-Württemberg Study are described. Measurements at level 1 "pupil" are characterized at first.

### Measurements at level 1 "pupil"

Measurements regarding the child's level are characterized in the following. All measurements were performed by four regional teams in fall 2010 and fall 2011 as described in section "Organization and Preparation of the Measurements". All measurements regarding the child's level were carried out in the classroom and gym of each primary school participating in the study.

#### Anthropometric Parameters

Measurements of the anthropometric parameters were performed according to the guidelines of the International Society for the Advancement of Kinanthropometry (ISAK). All examinations were performed by trained examiners in small groups of children separated by sex. Measurement of body weight was performed using calibrated flat scales (model 826, Seca^® ^Company, Germany). Mobile stadiometers (model 217, Seca^® ^Company, Germany) were used to measure body height. Waist circumference was measured halfway between the lower costal border and the iliac crest using a metal tape measure (Lufkin^® ^model W606PM, Lufkin Industries Inc., Texas, USA). Skinfold thickness was measured using calibrated Harpenden calipers (Baty International, Burgess Hill, UK) on the right hand side of the body. Triceps skinfold was measured halfway between the acromion border and the head of the radius on the back of the arm, subscapular skinfold was measured 2 cm down and out from the tip of the scapula.

#### Motor abilities

The Dordel-Koch-Test (DKT) [41,42] was performed by skilled examiners. The DKT is a test battery to assess the child's motor abilities including lateral jumps, sit and reach, standing long jump, sit-up, one-leg stand, push-ups, and a 6-min run.

#### General well-being and body image

General well-being was evaluated using an adapted Smilie-test [43] and body image was evaluated using the Figure Rating Scale [44] during a 1:1 interview, performed by trained examiners.

#### Mental abilities

Assessment of mental abilities was done using a computer-based test battery for attention and executive function (KiTAP) [45]. Three tasks from the KiTAP were used to assess the following mental abilities in the Baden-Württemberg Study: inhibition control, sustained attention, and mental flexibility. Number of errors and response time were measured for each task. The tests were performed by trained examiners in small groups using laptops with equivalent screen sizes. Each examiner tested 1 or 2 children at a time. Mental abilities were investigated in a subsample of the Baden-Württemberg Study.

#### Objective measurements of physical activity using accelerometry

Objective measurements of physical activity were performed using the Actiheart^® ^activity sensor (CamNtech, Cambridge, UK; The Actiheart User Manual 4.0.35. http://www.camntech.com/index.php). Activity sensors were worn continuously over a period of at least four to six successive days (two weekend days and two to four weekdays were included). Heart rate (beats per minute) and bodily acceleration (counts per minute) were recorded at 15 second intervals. Objective measurements of physical activity were performed in a subsample of the Baden-Württemberg Study.

### Measurements at level 2 "parents"

In the following section, details of all measurements on the parental level are described. The variables were assessed using a parental questionnaire.

#### Health-related quality of life

Health-related quality of life was assessed using two different instruments: the KINDL^®^-questionnaire (KINDL^® ^Manual [46]) and a visual analogue scale. Concerning the KINDL^® ^the 24 item-version for external assessment (in German) was used. Health-related quality of life was also assessed using the visual analogue scale (VAS) of the EQ-5D-Y questionnaire [47]. A proxy - version was used in the Baden-Württemberg Study: parents had to assess the child's health-related quality of life from their child's perspective.

#### Emotional fitness

Emotional fitness was assessed using the Children's Behaviour Questionnaire (CBQ) [48]. In the long version the CBQ includes 195 items assessing 15 scales. The short version of the CBQ includes 94 items assessing the same scales. For the Baden-Württemberg Study a selection of 55 items from the short version assessing 9 scales was made: activity level, impulsivity, anger/frustration, fear, sadness, soothability, smiling and laughter, attentional focusing, inhibitory control.

#### Socio-demographic variables

The following socio-demographic variables were asked in the parental questionnaire: the child's sex and date of birth, household income, size of flat, number of persons living in the household, number of working hours, employment status, educational level, and level of parents' professional training.

#### Further variables

Further variables asked in the parental questionnaire are, for instance, the variables for the child's anamnesis, risk factors for development of childhood overweight, child's behavior regarding nutrition, physical activity, and electronic media usage, as well as parental behavior regarding nutrition, physical activity, and electronic media usage and parents' attitudes to health.

### Measurements at level 3 "teacher and principal"

In the following, details of all measurements regarding the level "teacher and principal" are described. These measurements were carried out at follow-up only.

Two different questionnaires for intervention group and control group were used: in both study groups, characteristics of each school were collected about the number of pupils and teachers, the number of pupils with migration background, the concept of the school (e.g. full-time school, school with mixed-grades), recess format, school building and school environment (e.g. safety of way to school, form of settlement).

### Organization and preparation of the measurements

Pre-tests were performed to practice the planned procedure of the assessments. At first, in spring 2010 pre-tests for the different measurements at the level "pupil" were performed in several primary schools and kindergartens (final year) to ensure the practicability and feasibility of the assessment tools in children just entered school. For training purposes further preliminary tests in primary schools were carried out by each regional team before starting baseline measurements in fall 2010 and before starting follow-up measurements in fall 2011. Further, three different versions of the parental questionnaire were tested in May 2010 and June 2010 at five primary schools. None of these mentioned schools were later involved in the study.

Data collection at the level of pupils was conducted from the end of the summer vacation until the beginning of the fall vacation in 2010 and in 2011, respectively. For practical reasons four regional teams were established for data collection. Each team consisted of about 10 persons (two scientists and about 8 trained student assistants). The scientists are members of the research group in Ulm, the student assistants were recruited in the respective region. The scientists were trained in measuring anthropometric parameters by external experts. In addition; they were trained in handling and using Actiheart^® ^activity sensors. Student assistants were trained by the scientists in performing the DKT, interviews, computer-based test with children, and collecting anthropometric data with the exception of skinfolds. Skinfold thickness was only measured by scientists of the research group. Each team was measuring pupils of one or two classes per day; however, each school was measured separately.

The parental questionnaires were issued in November 2010 and November 2011, respectively, and returned within six weeks. Teachers and principals completed questionnaires at the second time in fall 2011.

### Recruitment process and randomization process

**Figure 2:**
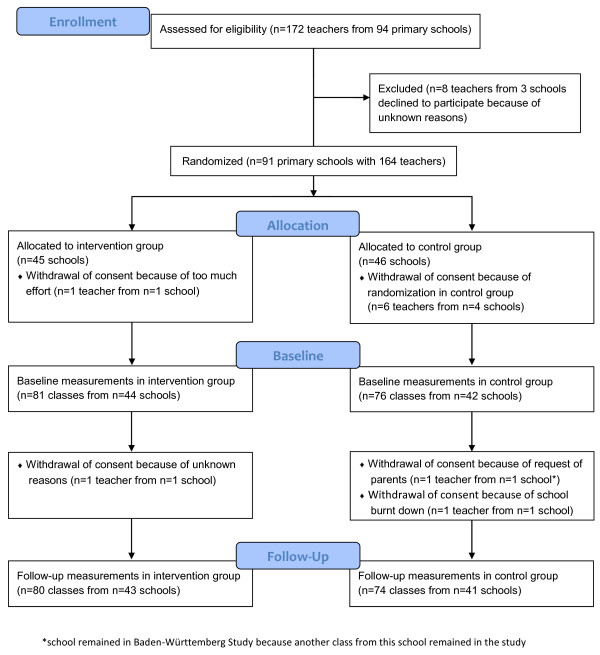
**Flow chart of enrollment, baseline measurements, and follow-up in the Baden-Württemberg Study**.

Information about the program and Baden-Württemberg Study were issued during the academic year 2009/2010 using a number of ways, e.g. education and health authorities, and universities of education; electronic newsletter; television and radio; adverts in training catalogs for primary school teachers; participation at trade shows. The recruitment process was also promoted by ten informative events in different parts of Baden-Württemberg. Further, all primary schools of the state of Baden-Württemberg received written information about the program and the structure of the study, asking teachers to participate. Interested teachers contacted the program center. The participation in the program was voluntary, participating teachers had to agree with randomization. In total, written consent from 172 primary school teachers and principals at 94 schools was obtained (see Figure 2). Teachers who already took part in the program in the academic year 2009/2010 were not included in the study. Because the grade level (1^st ^grade vs. 2^nd ^grade) may play a key role for the efficacy assessment, stratification of randomization was carried out for grade level according to the following procedure. Based on information about the distribution of the 172 teachers within the 94 schools, stratification according to number of classes and grade level (grade) was realized for the randomization process: six different school types were identified and used as strata for randomization:

• Primary schools with only one class (only one 1^st ^grade)

• Primary schools with only one class (only one 2^nd ^grade)

• Primary schools with more than one class (only 1^st ^grades)

• Primary schools with more than one class (only 2^nd ^grades)

• Primary schools with more than one class (1^st ^grades and 2^nd ^grades)

• Primary schools with mixed-grade classes (i.e. classes with pupils from 1^st ^and 2^nd ^grade)

There were 8 teachers at 3 schools who withdrew consent before randomization because of unknown reasons. Thus, randomization was performed for 164 teachers within 91 schools intervention group (IG): n = 45 schools, control group (CG): n = 46 schools). Written consent was withdrawn from 7 teachers within 5 schools immediately after randomization (IG: n = 1 school (reason: too much effort), CG: n = 4 schools (reason: randomization in control group)). Therefore, 86 schools (IG: n = 44 schools (n = 81 classes), CG: n = 42 schools (n = 76 classes)) were included in the Baden-Württemberg Study at baseline. The result of the stratified randomization was the following distribution of classes within both study groups:

• Intervention group: 1^st ^grade classes: n = 39, 2^nd ^grade classes: n = 32, mixed-grade classes: n = 10

• Control group: 1^st ^grade classes: n = 36, 2^nd ^grade classes: n = 29, mixed-grade classes: n = 11

Baseline measurements were conducted in 157 classes (i.e. 157 primary school teachers were included). One teacher at a school randomized in a 1^st ^grade class in the control group withdrew the consent shortly before follow-up (reason: parents' request). However, that school remains in the Baden-Württemberg Study because another class at that school took further part in the measurements at follow-up. Another two teachers randomized in a 1^st ^grade class in the control and intervention group, respectively withdrew consent at follow-up (reasons: school building burnt down and unknown). Thus, at follow-up the distribution of classes within both study groups was as follows:

• Intervention group: 1^st ^grade classes: n = 38, 2^nd ^grade classes: n = 32, mixed-grade classes: n = 10

• Control group: 1^st ^grade classes: n = 34, 2^nd ^grade classes: n = 29, mixed-grade classes: n = 11

In total, follow-up measurements were taken in 154 classes within 84 schools.

### Study sample at baseline

In total, 3159 pupils were in the participating classes and 1968 parents (62.3%) of pupils at primary schools participating in the Baden-Württemberg Study gave written informed consent to participate.

Baseline measurements (fall 2010) were taken from 1947 children and data at all three levels ("teacher and principal", "parents", and "pupil") was available from 1670 children (IG: n = 916 (54.9%), boys: n = 452 (49.3%); CG: n = 754 (45.1%), boys: n = 388 (51.5%)). The distribution of these pupils within each grade is shown in Table 1:

**Table 1:** Baseline measurement: Distribution of pupils of which data at all three levels are available (IG: intervention group, CG: control group)

grade	group	n (%)	% of boys
**1**	IG	411 (53.4%)	50.1%
	
	CG	358 (46.6%)	50.6%

**2**	IG	382 (55.5%)	45.6%
	
	CG	306 (44.5%)	50.3%

**mixed-grade classes (1/2)**	IG	123 (57.7%)	58.5%
	
	CG	90 (42.3%)	58.9%

The computer-based test for attention and executive function (KiTAP) were performed in a subsample of 513 pupils belonging to 45 classes within 27 schools (IG: 54.8%; boys: 50.3%), objective measurements of physical activity using Actiheart^® ^were performed in 384 pupils belonging to 56 classes within 32 schools (IG: 55.2%; boys: 50.3%). Results of baseline measurements in both subsamples are presented elsewhere [49,50].

Figure 2 shows the flow chart of enrollment, baseline measurements, and follow-up in the Baden-Württemberg Study.

### Power considerations

Power calculations were made at beginning of the study based on the availability of data for the main outcome parameters at baseline. Three main outcome parameters for the assessment of efficacy are specified in the study protocol: change in waist circumference, change in subscapular skinfold thickness and change in 6-min run performance, changes are calculated as differences between follow-up measurement and baseline measurement. All power calculations are based on simulations of hypothetic data and application of linear mixed effects models [51,52]. An adjustment according to Bonferroni was made to account for multiple testing [53]. In each mixed effects regression model only group (intervention group vs. control group) was used as explanatory variable and no distinction was made between several classes within the same school. Data from the project URMEL-ICE were used for specifying the differences between intervention group and control group. Randomization data were used for specifying the numbers of schools: IG: n = 44 schools, CG: n = 42 schools. Numbers of baseline measurements available for each primary outcome were used for calculation of pupil numbers in each study group:

• waist circumference: n = 1893 (IG: n = 1038, CG: n = 855)

• subscapular skinfold thickness: n = 1855 (IG: n = 1014, CG: n = 841)

• change in 6-min run performance: n = 1884 (IG: n = 1034, CG: n = 850)

Using this information, the following assumptions were made for the number of pupils in each school:

• change in waist circumference: IG: n = 24 (~1038 pupils in 44 schools); CG: n = 20 (about 855 pupils in 42 schools)

• change in subscapular skinfold thickness: IG: n = 23 (~1014 pupils in 44 schools); CG: n = 20 (about 841 pupils in 42 schools)

• change in 6-min run performance: IG: n = 24 (~1034 pupils in 44 schools); CG: n = 20 (about 850 pupils in 42 schools)

Furthermore, it was assumed that a percentage of 80% measurements available at baseline will be available at the follow-up, too. This percentage is an approximate value and was observed in the project URMEL-ICE.

The simulation results showed that the following values for statistical power could be reached if the assumptions proved right: 85% (change in waist circumference), 76% (change in subscapular skinfold thickness), and 74% (change in 6-min run performance).

### Statistical considerations

Besides descriptive methods, complex statistical models will be used for statistical analysis of main and further outcomes because of the clustered data structure. For example, mixed effects regression models [52] or GEE models [54] will be used. Both procedures can be extended in an easy way to adjust for possible covariates, e.g. variables regarding the familial and social background of children or time span between time of baseline measurement and time of follow-up measurement.

Health-economic aspects will be evaluated in a similar way as performed for the project URMEL-ICE [37].

## Discussion

"Komm mit in das gesunde Boot - Grundschule" is the largest health promotion program for primary school children in Germany. One advantage is that the program pursues the aims and contents of the existing curriculum and supports school teacher with easy useable material and information. The intervention consists of units incorporated during lessons and recess. The program expands the former URMEL-ICE study approach [15,16,37] from the 2^nd ^grade to all grades of the primary school and to the whole state of Baden-Württemberg. This makes the dissemination process crucial. The research team decided to use a system of project delivery consultants which are educated at the program center and disseminate information and material and provide courses in their region. The dissemination process is very successful: the program is implemented in 427 primary schools across Baden-Württemberg and 890 primary school teachers took part in local training courses performed by the project delivery consultants in the academic years 2009/2010 and 2010/2011.

The program is easily feasible by the class teacher and the additional effort required is very low. No external experts and no additional physical education lessons are necessary. An important point is the investigation of efficacy of the intervention which is part of the program: for a lot of other prevention programs efficacy could not be shown [27,28]. For an evaluation of the efficacy of the intervention the Baden-Württemberg Study is performed. It is a prospective, stratified, cluster-randomized, longitudinal study with two groups. Stratified randomization was performed for primary schools in Baden-Württemberg, South West Germany, according to school type (number of classes and grade). Despite withdrawal of written consent from 7 teachers of 5 schools, there is an approximately balanced number of classes in intervention and control group at baseline: within 1^st ^grade classes (39:36), within 2^nd ^grade classes (32:29), and within mixed-grade classes (10:11). Because only three teachers refused written consent after baseline measurements, the approximate balance between schools remained stable at follow-up. At baseline the percentage of boys in both study groups is comparable within 1^st ^grade classes (50.1% and 50.6%) and within mixed-grade classes (58.5% and 58.9%). A difference in this percentage is observed within 2^nd ^grade classes (45.6% and 50.3%). Therefore, an adjustment for sex will be realized in the statistical analysis.

The Baden-Württemberg Study has numerous important methodological strengths: a cluster-randomized controlled design (waiting control group design) was implemented with an intervention group and a control group. Stratified randomization was performed and a balanced ratio of classes in intervention group and in control group could be reached and even maintained at follow-up. The Baden-Württemberg Study is one of the largest studies in Germany to investigate the efficacy of an overweight and obesity prevention program and to promote the physical, mental, and emotional abilities: baseline measurements were taken of 1947 pupils; about 80% of this number is expected to take part in the follow-up measurements. Thus, intervention effects can be investigated in subgroups, e.g. girls vs. boys, children with vs. children without migration background. Efficacy will be investigated with comparatively objective main outcomes. Power simulations showed that adequate power for an investigation of the three main outcomes will be maintained. Furthermore, the study cohort is large enough for an adjustment for possible covariates, e.g. variables regarding the familial and socio-economic background of children or time span between time of baseline measurement and time of follow-up measurement.

However, there are some limitations of the Baden-Württemberg Study. First of all, blinding is not feasible in such studies and therefore a bias in the assessment of main and further outcomes cannot be ruled out (especially with subjective outcomes). Furthermore, the control groups are subject to public awareness to the health topics and teachers are motivated which is part of the study commitment which contaminates study effects and decreases power. The program's intervention is a low threshold procedure. Thus, effects of intervention might only be measurable after a longer study period and the duration of the intervention (one academic year) could also be too short to show effects.

Moreover, children and parents with low socio-economic background or migration background might not be reached by the program due to language and social barriers. However, the program itself has an integrative and inclusive approach which focuses on the whole school class including all children with different abilities, all risk groups, different social background and minorities. Informative material for parents was translated into the languages of the two dominant groups of immigrants in Germany (Turkish and Russian). However, children and parents with low socio-economic background or migration background could not be represented adequately in the Baden-Württemberg Study itself because of lower rate of informed consent or lower understanding of the questionnaires. This effect cannot be quantified, although the proportion of migrants seems to be not different from the total population (data not shown). Furthermore, a lot of variables (especially in the parental questionnaire) are assessed subjectively and a check of plausibility is very limited. Objective measurement of physical activity using Actiheart^® ^and assessment of mental abilities using KiTAP are limited to two subsamples because of organizational, technical, and time reasons. The analysis of baseline data shows that in each subsample the distribution of sex, educational level, and intervention group is comparable with the whole population in the Baden-Württemberg Study (not shown).

The research team anticipates that not only efficacy regarding the main outcomes will be shown but positive effects concerning further health related outcomes will occur. It is also anticipated that this study contributes to answering questions regarding the possibilities and limits of school-based programs and about the conditions and consequences of health promotion in pupils. There are examples for initial effective short-term prevention programs that could not maintain their success at later follow-ups [55,56]. Over the course of the years effects attenuated or were restricted to subgroups. Thus, there is a need for prevention programs which run long term and can be applied comprehensively, in spite of schools' limited financial and personnel resources and may be applicable in a large scale at state or country level.

Therefore, a continuation of the Baden-Württemberg Study is planned as observational follow-up study: the pupils taking part in the study will be observed at least until they finish primary school. Measurements are planned in the periods from end of summer vacation until fall vacation in the respective academic years, i.e. at annual intervals. Long-term effects of the intervention of the program "Komm mit in das gesunde Boot - Grundschule" could be investigated this way.

## Competing interests

The authors declare that they have no competing interests.

## Authors' contributions

JD and BK were involved in design and writing the study protocol of the Baden-Württemberg Study and drafted the manuscript. TW, AS, DK, OW, SKO, SKE, VH, MK, MW, SB, RM, TS, and JMS were involved in design and writing the study protocol and critically revised the manuscript. TW, AS, BK, DK, OW, SKO, SKE, DP, VH, MK, SB, and NF organized the Baden-Württemberg study, and were involved in carrying out the measurements. JD, MW and SS were involved in performing statistical evaluations and the power analysis. JMS is the director of the program "Komm mit in das gesunde Boot - Grundschule" and principal investigator of the Baden-Württemberg Study. The research team planned and organized the Baden-Württemberg Study, contributed to specific assessments and were partly involved in the performance of the measurements. All authors listed provided comments on the drafts and have read and approved the final version of this manuscript.

## Members of the advisory board

The members of the scientific advisory board are the following designated scientists: Prof. Dr. Ralph Beneke (Marburg), Prof Dr. Dr. Christine Graf (Cologne), Prof. Dr. Thomas Kohlmann (Greifswald), Prof. Dr. Renate Oberhoffer (Munich), and Prof. Dr. Petra Warschburger (Potsdam). No member belongs to the research group, no member is involved in the program.

## Pre-publication history

The pre-publication history for this paper can be accessed here:

http://www.biomedcentral.com/1471-2458/12/157/prepub
